# Supplementary material for: End Sequence Analysis Toolkit (ESAT) expands the extractable information from single-cell RNA-seq data
Source: Genome Res. 2016 Oct;26(10):1397–410. doi: 10.1101/gr.207902.116 (PMC5052061; doi:10.1101/gr.207902.116)
Supplement: Supplemental Material [file supp_gr.207902.116_Supp_Materials.tar.gz › ESAT-esat_v0.1/doc/html/classbroad_1_1pda_1_1seq_1_1segmentation_1_1_alignment_data_model_stats.html]

ESAT: broad.pda.seq.segmentation.AlignmentDataModelStats Class Reference


ESAT


- Main Page
- Related Pages
- Packages
- Classes
- Files

- Class List
- Class Index
- Class Hierarchy
- Class Members

All Classes Namespaces Files Functions Variables Enumerator Pages

- broad
- pda
- seq
- segmentation
- AlignmentDataModelStats

Classes |
Public Member Functions |
List of all members

broad.pda.seq.segmentation.AlignmentDataModelStats Class Reference

|  |  |
| --- | --- |
| Classes | |
| class | **ReadCountNormalizerToBaselineAlignment** |
|  | |

|  |  |
| --- | --- |
| Public Member Functions | |
|  | AlignmentDataModelStats () |
|  | |
|  | AlignmentDataModelStats (AlignmentDataModel data, Map< String, Integer > maskedRegions, String chr) throws IOException |
|  | |
|  | AlignmentDataModelStats (AlignmentDataModel data, Map< String, Integer > maskedRegions, String chr, double lambda, boolean loadChromosomeStats) throws IOException |
|  | |
|  | AlignmentDataModelStats (AlignmentDataModel data, Map< String, Integer > maskedRegions) throws IOException |
|  | |
|  | AlignmentDataModelStats (AlignmentDataModel data, Map< String, Integer > maskedRegions, Map< String, Double > readsPerChromosome) throws IOException |
|  | |
|  | AlignmentDataModelStats (AlignmentDataModel data, Map< String, Integer > maskedRegions, String chr, boolean loadChromosomeStats) throws IOException |
|  | |
|  | AlignmentDataModelStats (AlignmentDataModel data, Map< String, Integer > maskedRegions, double lambda) throws IOException |
|  | |
|  | AlignmentDataModelStats (AlignmentDataModel data, Map< String, Integer > maskedRegions, double lambda, boolean loadChromosomeStats) throws IOException |
|  | |
|  | AlignmentDataModelStats (AlignmentDataModel data, Map< String, Integer > maskedRegions, String chr, double lambda) throws IOException |
|  | |
|  | AlignmentDataModelStats (AlignmentDataModel data, String chr) throws IOException |
|  | |
|  | AlignmentDataModelStats (AlignmentDataModel data) throws IOException |
|  | |
| void | init (Map< String, Integer > maskedRegions, double lambda, boolean loadChromosomeStats) throws IOException |
|  | |
| void | init (Map< String, Integer > maskedRegions, double lambda, boolean loadChromosomeStats, Map< String, Double > readsByChr) throws IOException |
|  | |
| AlignmentDataModel | getData () |
|  | |
| double | getGlobalRPKMConstant () |
|  | |
| Map< String, Double > | getNumberOfReads () |
|  | |
| double | getLambda (String chr) throws IOException |
|  | |
| double | getSum (String chr) throws IOException |
|  | |
| double | getNumberOfReads (String chr) throws IOException |
|  | |
| double | getNumberOfReadsByChr (String chr) throws IOException |
|  | |
| int | getTotalReads () throws IOException |
|  | |
| double | getNumberMarkers (String chr) throws IOException |
|  | |
| Map< String, Integer > | getChromosomeLengths () |
|  | |
| int[] | getGeneCounts (Collection< Gene > genes, int extensionFactor) throws IOException |
|  | |
| int[] | getGeneCountsWithoutDatasetTotals (Collection< Gene > genes, int extensionFactor) throws IOException |
|  | |
| Map< Gene, double[]> | getGeneExpression (Collection< Gene > genes, int extensionFactor) throws IOException |
|  | |
| int | getCounts (Collection< Annotation > Annotation, int extensionFactor) throws IOException |
|  | |
| double | getCountsPerAlignment (Annotation align, IntervalTree< Alignment > tree, int extensionFactor) |
|  | |
| double | getCountsPerAlignment (Gene window, IntervalTree< Alignment > tree, int extensionFactor) |
|  | |
| double | getCountsPerAlignment (Annotation align, Map< String, IntervalTree< Annotation >> exonTree, IntervalTree< Alignment > tree, int extensionFactor) |
|  | |
| double | getRPKMConstant (String chr) |
|  | |
| Map< Annotation, Integer > | getSplicedReads (Annotation chrRegion, int i, int j) throws IOException |
|  | |
| Map< Annotation, Integer > | getSplicedReads (Annotation region, List< Predicate< Annotation >> filters, int minNumIntrons) throws IOException |
|  | |
| Map< Annotation, Integer > | getSplicedReads (Annotation region, int minIntronSize, int maxIntronSize, int minNumberOfSplices, Sequence chrSequence) throws IOException |
|  | |
| IntervalTree< Alignment > | getIntervalTree (LightweightGenomicAnnotation region) throws IOException |
|  | |
| IntervalTree< Alignment > | getIntervalTree (String chr, int start, int end) throws IOException |
|  | |
| IntervalTree< Alignment > | getIntervalTreeCached (String chr, int start, int end) throws IOException |
|  | |
| IntervalTree< Alignment > | getIntervalTreeTruncatedCached (String chromosome, int start, int end) throws IOException |
|  | |
| double | getScorePerAlignmentFromCache (Gene window, IntervalTree< Alignment > chunkAlignmentTree, int extensionFactor) |
|  | |
| int | getChunkStart () |
|  | |
| int | getChunkEnd () |
|  | |
| double | getSpliceWeightFactor () |
|  | |
| Iterator< Annotation > | getExonAnnotationOverlappingRegion (Annotation align) throws IOException |
|  | |
| CloseableIterator< Alignment > | getAnnotationOverlappingRegion (Annotation Annotation) throws IOException |
|  | |
| double | getCountsOfUniqueOverlappers (Annotation startPosition, Annotation window, IntervalTree< Alignment > chunkAlignmentTree, int extensionFactor) |
|  | |
| CloseableIterator< Alignment > | getReadIterator () |
|  | |
| CloseableIterator< Alignment > | getReadIterator (Annotation region) throws IOException |
|  | |
| double | getMinimumMappingQuality () |
|  | |
| void | setAlignmentDataModel (AlignmentDataModel data) |
|  | |
| void | setCountNormalizer (ReadCountNormalizer normalizer) |
|  | |
| void | setLambda (String chr, double numberOfReads) |
|  | |
| void | setNumberOfReads (String chr, double numberOfReads2) |
|  | |
| void | setMinimumMappingQuality (double minMapQual) |
|  | |
| void | setNumberOfMarkers (String chr, double numMarkers2) |
|  | |
| void | setChunkSize (int chunkSize) |
|  | |
| void | resetTreeCache () |
|  | |
| void | resetTreeCache (String chr) |
|  | |
| double[] | scanPRate (Gene gene, int extensionFactor) throws IOException |
|  | |
| double[] | scanRegion (int fixedWidth, LightweightGenomicAnnotation region) throws IOException |
|  | |
| Gene | getPeak (Gene gene, Gene startCodon, IntervalTree< Alignment > tree, int EF) |
|  | |
| double[] | scanPRate (LightweightGenomicAnnotation annotation, int extensionFactor) throws IOException |
|  | |
| double[] | scanPRate (Gene gene, IntervalTree< Alignment > tree, int extensionFactor) throws IOException |
|  | |
| double[] | scanPRate (Annotation align, IntervalTree< Alignment > tree, double localLambda, int extensionFactor) throws IOException |
|  | |
| double[] | scanPRate (Annotation align, double readCount, int extenstionFactor) throws IOException |
|  | |
| double[] | scanPRate (Annotation align, double readCount, double localLambda, int extenstionFactor) throws IOException |
|  | |
| double[] | scanPRate (Gene gene, IntervalTree< Alignment > tree, double localLambda, int extensionFactor) throws IOException |
|  | |
| double[] | scanPRate (Gene gene, IntervalTree< Alignment > tree, double localLambda, int extensionFactor, double numMarkers) throws IOException |
|  | |
| double | count (Annotation align, int extensionFactor) throws IOException |
|  | |
| int | countWithinExon (Annotation align, int extensionFactor) throws IOException |
|  | |
| boolean | isPaired () |
|  | |
| int | chromosomeLength (String chr) |
|  | |
| boolean | passes (Gene window, IntervalTree< Alignment > chunkAlignmentTree, int i, int critVal) |
|  | |
| void | normalizeReadCountsToBaseModel (AlignmentDataModelStats baseline) throws IOException |
|  | |
| double | getMaskedRegionsSize (String chr) |
|  | |
| void | setStoredStats (StoredAlignmentStats sas) |
|  | |
| void | setExtensionFactor (int extensionFactor) |
|  | |

## Constructor & Destructor Documentation

|  |  |  |  |  |
| --- | --- | --- | --- | --- |
| broad.pda.seq.segmentation.AlignmentDataModelStats.AlignmentDataModelStats | ( |  | ) |  |

|  |  |  |  |
| --- | --- | --- | --- |
| broad.pda.seq.segmentation.AlignmentDataModelStats.AlignmentDataModelStats | ( | AlignmentDataModel | *data*, |
|  |  | Map< String, Integer > | *maskedRegions*, |
|  |  | String | *chr* |
|  | ) |  | throws IOException |

|  |  |  |  |
| --- | --- | --- | --- |
| broad.pda.seq.segmentation.AlignmentDataModelStats.AlignmentDataModelStats | ( | AlignmentDataModel | *data*, |
|  |  | Map< String, Integer > | *maskedRegions*, |
|  |  | String | *chr*, |
|  |  | double | *lambda*, |
|  |  | boolean | *loadChromosomeStats* |
|  | ) |  | throws IOException |

Here is the call graph for this function:

|  |  |  |  |
| --- | --- | --- | --- |
| broad.pda.seq.segmentation.AlignmentDataModelStats.AlignmentDataModelStats | ( | AlignmentDataModel | *data*, |
|  |  | Map< String, Integer > | *maskedRegions* |
|  | ) |  | throws IOException |

Here is the call graph for this function:

|  |  |  |  |
| --- | --- | --- | --- |
| broad.pda.seq.segmentation.AlignmentDataModelStats.AlignmentDataModelStats | ( | AlignmentDataModel | *data*, |
|  |  | Map< String, Integer > | *maskedRegions*, |
|  |  | Map< String, Double > | *readsPerChromosome* |
|  | ) |  | throws IOException |

Here is the call graph for this function:

|  |  |  |  |
| --- | --- | --- | --- |
| broad.pda.seq.segmentation.AlignmentDataModelStats.AlignmentDataModelStats | ( | AlignmentDataModel | *data*, |
|  |  | Map< String, Integer > | *maskedRegions*, |
|  |  | String | *chr*, |
|  |  | boolean | *loadChromosomeStats* |
|  | ) |  | throws IOException |

|  |  |  |  |
| --- | --- | --- | --- |
| broad.pda.seq.segmentation.AlignmentDataModelStats.AlignmentDataModelStats | ( | AlignmentDataModel | *data*, |
|  |  | Map< String, Integer > | *maskedRegions*, |
|  |  | double | *lambda* |
|  | ) |  | throws IOException |

Here is the call graph for this function:

|  |  |  |  |
| --- | --- | --- | --- |
| broad.pda.seq.segmentation.AlignmentDataModelStats.AlignmentDataModelStats | ( | AlignmentDataModel | *data*, |
|  |  | Map< String, Integer > | *maskedRegions*, |
|  |  | double | *lambda*, |
|  |  | boolean | *loadChromosomeStats* |
|  | ) |  | throws IOException |

Here is the call graph for this function:

|  |  |  |  |
| --- | --- | --- | --- |
| broad.pda.seq.segmentation.AlignmentDataModelStats.AlignmentDataModelStats | ( | AlignmentDataModel | *data*, |
|  |  | Map< String, Integer > | *maskedRegions*, |
|  |  | String | *chr*, |
|  |  | double | *lambda* |
|  | ) |  | throws IOException |

|  |  |  |  |
| --- | --- | --- | --- |
| broad.pda.seq.segmentation.AlignmentDataModelStats.AlignmentDataModelStats | ( | AlignmentDataModel | *data*, |
|  |  | String | *chr* |
|  | ) |  | throws IOException |

|  |  |  |  |  |  |
| --- | --- | --- | --- | --- | --- |
| broad.pda.seq.segmentation.AlignmentDataModelStats.AlignmentDataModelStats | ( | AlignmentDataModel | *data* | ) | throws IOException |

Here is the call graph for this function:

## Member Function Documentation

|  |  |  |  |  |  |
| --- | --- | --- | --- | --- | --- |
| int broad.pda.seq.segmentation.AlignmentDataModelStats.chromosomeLength | ( | String | *chr* | ) |  |

|  |  |  |  |
| --- | --- | --- | --- |
| double broad.pda.seq.segmentation.AlignmentDataModelStats.count | ( | Annotation | *align*, |
|  |  | int | *extensionFactor* |
|  | ) |  | throws IOException |

Here is the caller graph for this function:

|  |  |  |  |
| --- | --- | --- | --- |
| int broad.pda.seq.segmentation.AlignmentDataModelStats.countWithinExon | ( | Annotation | *align*, |
|  |  | int | *extensionFactor* |
|  | ) |  | throws IOException |

|  |  |  |  |  |  |
| --- | --- | --- | --- | --- | --- |
| CloseableIterator<Alignment> broad.pda.seq.segmentation.AlignmentDataModelStats.getAnnotationOverlappingRegion | ( | Annotation | *Annotation* | ) | throws IOException |

|  |  |  |  |  |
| --- | --- | --- | --- | --- |
| Map<String, Integer> broad.pda.seq.segmentation.AlignmentDataModelStats.getChromosomeLengths | ( |  | ) |  |

Here is the caller graph for this function:

|  |  |  |  |  |
| --- | --- | --- | --- | --- |
| int broad.pda.seq.segmentation.AlignmentDataModelStats.getChunkEnd | ( |  | ) |  |

|  |  |  |  |  |
| --- | --- | --- | --- | --- |
| int broad.pda.seq.segmentation.AlignmentDataModelStats.getChunkStart | ( |  | ) |  |

|  |  |  |  |
| --- | --- | --- | --- |
| int broad.pda.seq.segmentation.AlignmentDataModelStats.getCounts | ( | Collection< Annotation > | *Annotation*, |
|  |  | int | *extensionFactor* |
|  | ) |  | throws IOException |

|  |  |  |  |
| --- | --- | --- | --- |
| double broad.pda.seq.segmentation.AlignmentDataModelStats.getCountsOfUniqueOverlappers | ( | Annotation | *startPosition*, |
|  |  | Annotation | *window*, |
|  |  | IntervalTree< Alignment > | *chunkAlignmentTree*, |
|  |  | int | *extensionFactor* |
|  | ) |  |  |

|  |  |  |  |
| --- | --- | --- | --- |
| double broad.pda.seq.segmentation.AlignmentDataModelStats.getCountsPerAlignment | ( | Annotation | *align*, |
|  |  | IntervalTree< Alignment > | *tree*, |
|  |  | int | *extensionFactor* |
|  | ) |  |  |

|  |  |  |  |
| --- | --- | --- | --- |
| double broad.pda.seq.segmentation.AlignmentDataModelStats.getCountsPerAlignment | ( | Gene | *window*, |
|  |  | IntervalTree< Alignment > | *tree*, |
|  |  | int | *extensionFactor* |
|  | ) |  |  |

|  |  |  |  |
| --- | --- | --- | --- |
| double broad.pda.seq.segmentation.AlignmentDataModelStats.getCountsPerAlignment | ( | Annotation | *align*, |
|  |  | Map< String, IntervalTree< Annotation >> | *exonTree*, |
|  |  | IntervalTree< Alignment > | *tree*, |
|  |  | int | *extensionFactor* |
|  | ) |  |  |

|  |  |  |  |  |
| --- | --- | --- | --- | --- |
| AlignmentDataModel broad.pda.seq.segmentation.AlignmentDataModelStats.getData | ( |  | ) |  |

|  |  |  |  |  |  |
| --- | --- | --- | --- | --- | --- |
| Iterator<Annotation> broad.pda.seq.segmentation.AlignmentDataModelStats.getExonAnnotationOverlappingRegion | ( | Annotation | *align* | ) | throws IOException |

|  |  |  |  |
| --- | --- | --- | --- |
| int [] broad.pda.seq.segmentation.AlignmentDataModelStats.getGeneCounts | ( | Collection< Gene > | *genes*, |
|  |  | int | *extensionFactor* |
|  | ) |  | throws IOException |

Here is the call graph for this function:

|  |  |  |  |
| --- | --- | --- | --- |
| int [] broad.pda.seq.segmentation.AlignmentDataModelStats.getGeneCountsWithoutDatasetTotals | ( | Collection< Gene > | *genes*, |
|  |  | int | *extensionFactor* |
|  | ) |  | throws IOException |

Get read counts mapping to exons, introns, 5'UTRs and 3'UTRs in gene set of interest

Parameters
:   |  |  |
    | --- | --- |
    | genes |  |
    | extensionFactor |  |

Returns
:   An int array consisting of counts for exons, introns, 5'UTRs, 3'UTRs

Exceptions
:   |  |  |
    | --- | --- |
    | IOException |  |

|  |  |  |  |
| --- | --- | --- | --- |
| Map<Gene, double[]> broad.pda.seq.segmentation.AlignmentDataModelStats.getGeneExpression | ( | Collection< Gene > | *genes*, |
|  |  | int | *extensionFactor* |
|  | ) |  | throws IOException |

Here is the call graph for this function:

|  |  |  |  |  |
| --- | --- | --- | --- | --- |
| double broad.pda.seq.segmentation.AlignmentDataModelStats.getGlobalRPKMConstant | ( |  | ) |  |

|  |  |  |  |  |  |
| --- | --- | --- | --- | --- | --- |
| IntervalTree<Alignment> broad.pda.seq.segmentation.AlignmentDataModelStats.getIntervalTree | ( | LightweightGenomicAnnotation | *region* | ) | throws IOException |

Here is the caller graph for this function:

|  |  |  |  |
| --- | --- | --- | --- |
| IntervalTree<Alignment> broad.pda.seq.segmentation.AlignmentDataModelStats.getIntervalTree | ( | String | *chr*, |
|  |  | int | *start*, |
|  |  | int | *end* |
|  | ) |  | throws IOException |

|  |  |  |  |
| --- | --- | --- | --- |
| IntervalTree<Alignment> broad.pda.seq.segmentation.AlignmentDataModelStats.getIntervalTreeCached | ( | String | *chr*, |
|  |  | int | *start*, |
|  |  | int | *end* |
|  | ) |  | throws IOException |

|  |  |  |  |
| --- | --- | --- | --- |
| IntervalTree<Alignment> broad.pda.seq.segmentation.AlignmentDataModelStats.getIntervalTreeTruncatedCached | ( | String | *chromosome*, |
|  |  | int | *start*, |
|  |  | int | *end* |
|  | ) |  | throws IOException |

|  |  |  |  |  |  |
| --- | --- | --- | --- | --- | --- |
| double broad.pda.seq.segmentation.AlignmentDataModelStats.getLambda | ( | String | *chr* | ) | throws IOException |

Here is the caller graph for this function:

|  |  |  |  |  |  |
| --- | --- | --- | --- | --- | --- |
| double broad.pda.seq.segmentation.AlignmentDataModelStats.getMaskedRegionsSize | ( | String | *chr* | ) |  |

|  |  |  |  |  |
| --- | --- | --- | --- | --- |
| double broad.pda.seq.segmentation.AlignmentDataModelStats.getMinimumMappingQuality | ( |  | ) |  |

|  |  |  |  |  |  |
| --- | --- | --- | --- | --- | --- |
| double broad.pda.seq.segmentation.AlignmentDataModelStats.getNumberMarkers | ( | String | *chr* | ) | throws IOException |

Here is the caller graph for this function:

|  |  |  |  |  |
| --- | --- | --- | --- | --- |
| Map<String, Double> broad.pda.seq.segmentation.AlignmentDataModelStats.getNumberOfReads | ( |  | ) |  |

|  |  |  |  |  |  |
| --- | --- | --- | --- | --- | --- |
| double broad.pda.seq.segmentation.AlignmentDataModelStats.getNumberOfReads | ( | String | *chr* | ) | throws IOException |

Here is the call graph for this function:

|  |  |  |  |  |  |
| --- | --- | --- | --- | --- | --- |
| double broad.pda.seq.segmentation.AlignmentDataModelStats.getNumberOfReadsByChr | ( | String | *chr* | ) | throws IOException |

Here is the call graph for this function:

|  |  |  |  |
| --- | --- | --- | --- |
| Gene broad.pda.seq.segmentation.AlignmentDataModelStats.getPeak | ( | Gene | *gene*, |
|  |  | Gene | *startCodon*, |
|  |  | IntervalTree< Alignment > | *tree*, |
|  |  | int | *EF* |
|  | ) |  |  |

|  |  |  |  |  |
| --- | --- | --- | --- | --- |
| CloseableIterator<Alignment> broad.pda.seq.segmentation.AlignmentDataModelStats.getReadIterator | ( |  | ) |  |

|  |  |  |  |  |  |
| --- | --- | --- | --- | --- | --- |
| CloseableIterator<Alignment> broad.pda.seq.segmentation.AlignmentDataModelStats.getReadIterator | ( | Annotation | *region* | ) | throws IOException |

|  |  |  |  |  |  |
| --- | --- | --- | --- | --- | --- |
| double broad.pda.seq.segmentation.AlignmentDataModelStats.getRPKMConstant | ( | String | *chr* | ) |  |

Here is the caller graph for this function:

|  |  |  |  |
| --- | --- | --- | --- |
| double broad.pda.seq.segmentation.AlignmentDataModelStats.getScorePerAlignmentFromCache | ( | Gene | *window*, |
|  |  | IntervalTree< Alignment > | *chunkAlignmentTree*, |
|  |  | int | *extensionFactor* |
|  | ) |  |  |

|  |  |  |  |
| --- | --- | --- | --- |
| Map<Annotation, Integer> broad.pda.seq.segmentation.AlignmentDataModelStats.getSplicedReads | ( | Annotation | *chrRegion*, |
|  |  | int | *i*, |
|  |  | int | *j* |
|  | ) |  | throws IOException |

|  |  |  |  |
| --- | --- | --- | --- |
| Map<Annotation, Integer> broad.pda.seq.segmentation.AlignmentDataModelStats.getSplicedReads | ( | Annotation | *region*, |
|  |  | List< Predicate< Annotation >> | *filters*, |
|  |  | int | *minNumIntrons* |
|  | ) |  | throws IOException |

|  |  |  |  |
| --- | --- | --- | --- |
| Map<Annotation, Integer> broad.pda.seq.segmentation.AlignmentDataModelStats.getSplicedReads | ( | Annotation | *region*, |
|  |  | int | *minIntronSize*, |
|  |  | int | *maxIntronSize*, |
|  |  | int | *minNumberOfSplices*, |
|  |  | Sequence | *chrSequence* |
|  | ) |  | throws IOException |

|  |  |  |  |  |
| --- | --- | --- | --- | --- |
| double broad.pda.seq.segmentation.AlignmentDataModelStats.getSpliceWeightFactor | ( |  | ) |  |

Here is the caller graph for this function:

|  |  |  |  |  |  |
| --- | --- | --- | --- | --- | --- |
| double broad.pda.seq.segmentation.AlignmentDataModelStats.getSum | ( | String | *chr* | ) | throws IOException |

Here is the caller graph for this function:

|  |  |  |  |  |
| --- | --- | --- | --- | --- |
| int broad.pda.seq.segmentation.AlignmentDataModelStats.getTotalReads | ( |  | ) | throws IOException |

Here is the call graph for this function:

Here is the caller graph for this function:

|  |  |  |  |
| --- | --- | --- | --- |
| void broad.pda.seq.segmentation.AlignmentDataModelStats.init | ( | Map< String, Integer > | *maskedRegions*, |
|  |  | double | *lambda*, |
|  |  | boolean | *loadChromosomeStats* |
|  | ) |  | throws IOException |

Here is the call graph for this function:

Here is the caller graph for this function:

|  |  |  |  |
| --- | --- | --- | --- |
| void broad.pda.seq.segmentation.AlignmentDataModelStats.init | ( | Map< String, Integer > | *maskedRegions*, |
|  |  | double | *lambda*, |
|  |  | boolean | *loadChromosomeStats*, |
|  |  | Map< String, Double > | *readsByChr* |
|  | ) |  | throws IOException |

Here is the call graph for this function:

|  |  |  |  |  |
| --- | --- | --- | --- | --- |
| boolean broad.pda.seq.segmentation.AlignmentDataModelStats.isPaired | ( |  | ) |  |

Here is the call graph for this function:

Here is the caller graph for this function:

|  |  |  |  |  |  |
| --- | --- | --- | --- | --- | --- |
| void broad.pda.seq.segmentation.AlignmentDataModelStats.normalizeReadCountsToBaseModel | ( | AlignmentDataModelStats | *baseline* | ) | throws IOException |

Here is the call graph for this function:

|  |  |  |  |
| --- | --- | --- | --- |
| boolean broad.pda.seq.segmentation.AlignmentDataModelStats.passes | ( | Gene | *window*, |
|  |  | IntervalTree< Alignment > | *chunkAlignmentTree*, |
|  |  | int | *i*, |
|  |  | int | *critVal* |
|  | ) |  |  |

|  |  |  |  |  |
| --- | --- | --- | --- | --- |
| void broad.pda.seq.segmentation.AlignmentDataModelStats.resetTreeCache | ( |  | ) |  |

|  |  |  |  |  |  |
| --- | --- | --- | --- | --- | --- |
| void broad.pda.seq.segmentation.AlignmentDataModelStats.resetTreeCache | ( | String | *chr* | ) |  |

|  |  |  |  |
| --- | --- | --- | --- |
| double [] broad.pda.seq.segmentation.AlignmentDataModelStats.scanPRate | ( | Gene | *gene*, |
|  |  | int | *extensionFactor* |
|  | ) |  | throws IOException |

Here is the call graph for this function:

Here is the caller graph for this function:

|  |  |  |  |
| --- | --- | --- | --- |
| double [] broad.pda.seq.segmentation.AlignmentDataModelStats.scanPRate | ( | LightweightGenomicAnnotation | *annotation*, |
|  |  | int | *extensionFactor* |
|  | ) |  | throws IOException |

Here is the call graph for this function:

|  |  |  |  |
| --- | --- | --- | --- |
| double [] broad.pda.seq.segmentation.AlignmentDataModelStats.scanPRate | ( | Gene | *gene*, |
|  |  | IntervalTree< Alignment > | *tree*, |
|  |  | int | *extensionFactor* |
|  | ) |  | throws IOException |

Here is the call graph for this function:

|  |  |  |  |
| --- | --- | --- | --- |
| double [] broad.pda.seq.segmentation.AlignmentDataModelStats.scanPRate | ( | Annotation | *align*, |
|  |  | IntervalTree< Alignment > | *tree*, |
|  |  | double | *localLambda*, |
|  |  | int | *extensionFactor* |
|  | ) |  | throws IOException |

Here is the call graph for this function:

|  |  |  |  |
| --- | --- | --- | --- |
| double [] broad.pda.seq.segmentation.AlignmentDataModelStats.scanPRate | ( | Annotation | *align*, |
|  |  | double | *readCount*, |
|  |  | int | *extenstionFactor* |
|  | ) |  | throws IOException |

Here is the call graph for this function:

|  |  |  |  |
| --- | --- | --- | --- |
| double [] broad.pda.seq.segmentation.AlignmentDataModelStats.scanPRate | ( | Annotation | *align*, |
|  |  | double | *readCount*, |
|  |  | double | *localLambda*, |
|  |  | int | *extenstionFactor* |
|  | ) |  | throws IOException |

Here is the call graph for this function:

|  |  |  |  |
| --- | --- | --- | --- |
| double [] broad.pda.seq.segmentation.AlignmentDataModelStats.scanPRate | ( | Gene | *gene*, |
|  |  | IntervalTree< Alignment > | *tree*, |
|  |  | double | *localLambda*, |
|  |  | int | *extensionFactor* |
|  | ) |  | throws IOException |

Here is the call graph for this function:

|  |  |  |  |
| --- | --- | --- | --- |
| double [] broad.pda.seq.segmentation.AlignmentDataModelStats.scanPRate | ( | Gene | *gene*, |
|  |  | IntervalTree< Alignment > | *tree*, |
|  |  | double | *localLambda*, |
|  |  | int | *extensionFactor*, |
|  |  | double | *numMarkers* |
|  | ) |  | throws IOException |

Here is the call graph for this function:

|  |  |  |  |
| --- | --- | --- | --- |
| double [] broad.pda.seq.segmentation.AlignmentDataModelStats.scanRegion | ( | int | *fixedWidth*, |
|  |  | LightweightGenomicAnnotation | *region* |
|  | ) |  | throws IOException |

Here is the call graph for this function:

|  |  |  |  |  |  |
| --- | --- | --- | --- | --- | --- |
| void broad.pda.seq.segmentation.AlignmentDataModelStats.setAlignmentDataModel | ( | AlignmentDataModel | *data* | ) |  |

|  |  |  |  |  |  |
| --- | --- | --- | --- | --- | --- |
| void broad.pda.seq.segmentation.AlignmentDataModelStats.setChunkSize | ( | int | *chunkSize* | ) |  |

|  |  |  |  |  |  |
| --- | --- | --- | --- | --- | --- |
| void broad.pda.seq.segmentation.AlignmentDataModelStats.setCountNormalizer | ( | ReadCountNormalizer | *normalizer* | ) |  |

Here is the caller graph for this function:

|  |  |  |  |  |  |
| --- | --- | --- | --- | --- | --- |
| void broad.pda.seq.segmentation.AlignmentDataModelStats.setExtensionFactor | ( | int | *extensionFactor* | ) |  |

|  |  |  |  |
| --- | --- | --- | --- |
| void broad.pda.seq.segmentation.AlignmentDataModelStats.setLambda | ( | String | *chr*, |
|  |  | double | *numberOfReads* |
|  | ) |  |  |

|  |  |  |  |  |  |
| --- | --- | --- | --- | --- | --- |
| void broad.pda.seq.segmentation.AlignmentDataModelStats.setMinimumMappingQuality | ( | double | *minMapQual* | ) |  |

|  |  |  |  |
| --- | --- | --- | --- |
| void broad.pda.seq.segmentation.AlignmentDataModelStats.setNumberOfMarkers | ( | String | *chr*, |
|  |  | double | *numMarkers2* |
|  | ) |  |  |

|  |  |  |  |
| --- | --- | --- | --- |
| void broad.pda.seq.segmentation.AlignmentDataModelStats.setNumberOfReads | ( | String | *chr*, |
|  |  | double | *numberOfReads2* |
|  | ) |  |  |

|  |  |  |  |  |  |
| --- | --- | --- | --- | --- | --- |
| void broad.pda.seq.segmentation.AlignmentDataModelStats.setStoredStats | ( | StoredAlignmentStats | *sas* | ) |  |

---

The documentation for this class was generated from the following file:

- java/broad/pda/seq/segmentation/AlignmentDataModelStats.java


---

Generated on Fri Aug 15 2014 15:11:04 for ESAT by  

 1.8.7
